# Stability Analysis of Delayed Immune Response BCG Infection in Bladder Cancer Treatment Model by Stochastic Perturbations

**DOI:** 10.1155/2018/9653873

**Published:** 2018-07-09

**Authors:** Leonid Shaikhet, Svetlana Bunimovich-Mendrazitsky

**Affiliations:** Department of Mathematics, Ariel University, Ariel 40700, Israel

## Abstract

We present a revised mathematical model of the immune response to Bacillus Calmette-Guérin (BCG) treatment of bladder cancer, optimized according to biological and clinical data accumulated during the last decade. The improved model accounts for cytotoxic T lymphocyte differentiation as an integral element of the delayed immune response, as well as the logistic growth terms for cancer cell proliferation. Three equilibria are demonstrated for the proposed model, which is assumed to be influenced by white noise stochastic perturbations that are directly proportional to the system state deviation from an equilibrium. Stability conditions for all equilibria are analyzed using the Kolmanovskii-Shaikhet general method of Lyapunov functionals construction.

## 1. Introduction

Bladder cancer (BC) is 7th most common cancer (the 4th most common for men) with approximately 356,000 new cases each year and more than 145,000 deaths per year. The highest incidence occurs in industrialized and developed areas such as Europe, North America, and Australia (Jemal et al., [1]). Tobacco smoking is the main BC risk factor, accounting for at least 50% of BC cases. Roughly 10% of all BC cases have been related to occupational exposure to chemicals and dye, mostly in industrial areas processing paint, metal, and petroleum products (Bunimovich-Mendrazitsky et al. [2]).

The treatment of the BC has improved during last 40 years due to development of high definition of cystoscopy, newly technology in the bladder drugs instillation. However, the prognosis of advanced bladder cancer has not improved during the last years (Alexandroff et al. [3]). The high rates of recurrence, invasive surveillance strategies, and high treatment costs combine to make bladder cancer the single most expensive cancer in both England and the United States (Eylert et al. [4]).

BC is most frequently treated with intravesical instillations of an adjuvant immunotherapy with the Bacillus Calmette-Guérin (BCG) bacteria. BCG immunotherapy, originally established by Morales et al. [5], is administered after surgical removal of the tumor at a point where no visual lesions or morphologically evident malignant cells present in random biopsies (Brandau and Suttman, [6]). BCG immunotherapy has proven its superiority over chemotherapy in reducing tumor recurrence rates for patients with high grade or high risk nonmuscle invasive BC. While Lamm et al. [7] found BCG to even reduce disease progression, there is a need to understand why the standard BCG treatment protocol is not effective for nonresponding or relapsing patients. The BCG treatment protocol remains to be optimized specifically for those patients who do not achieve remission from treatment with the standard regimen.

In last three decades, it has been commonly accepted that a qualitative understanding of dynamic cancer treatment requires a mathematical framework in which the essential features of this complex process are represented (Byrne, [8]). The model proposed in [9] was the first mathematical model to describe tumor-immune system interactions in the bladder as a result of continuous BCG therapy. Bunimovich-Mendrazitsky et al. [9–12] have modeled the use of BCG in noninvasive bladder cancer, identifying stability points of the mathematical system to ensure durability of the simulated results, and found a tolerable bacteria threshold and effective treatment regimens to minimize undesirable side effects. A system of ordinary differential equations (ODE) was used for effective description of BCG treatment dynamics. In this manuscript, we present an improved BCG model based upon that of B-M et al. [9] which describes the tumor-immune system interactions in the bladder in response to BCG therapy, updated according to newly published biological and clinical data. Three equilibria for the optimized model are demonstrated.

One of the main problems encountered in mathematical models described by differential equations is that of their stability. In this work, equilibria stability is analyzed using the Kolmanovskii-Shaikhet general method of Lyapunov functionals construction [13–17] and the method of linear matrix inequalities (LMIs). Equilibria stability in probability of a system of nonlinear stochastic differential equations possessing the order of nonlinearity higher than one can be reduced to investigation of asymptotic mean square stability of the linear part of the considered nonlinear system. The proposed method of stability investigation is based upon a preliminary approximation in which the nonlinear system is centralized and linearized around an equilibrium point, and the zero solution of the obtained linear system is investigated for asymptotic mean square stability. Obtained asymptotic mean square stability conditions of the linear system zero solution at the same time are sufficient conditions for stability in probability of a corresponding equilibrium of the initial nonlinear system. This method can be applied to a system of arbitrary nonlinear differential equations with the order of nonlinearity higher than one.

The manuscript is organized as follows: in Section 2, we give general information about the BC and the biological background on which our model is based. The mathematical derivation of the improved model is described in Section 3. In Section 4 we discuss the existence of three equilibria.  Section 5 is devoted to stochastic perturbations, centering, and linearization of the considered system around equilibria including description of an appropriate linear system for each from three equilibria. The conditions of stability for all three equilibria and some numerical examples are discussed in Section 6. Some auxiliary information is presented in Appendix. Conclusions from a number of clinical scenarios leading to successful treatment or to progression of cancer and some future projects are outlined in Section 7.

## 2. Biological Framework for Optimization of the Model

BCG is thought to encourage tumor elimination by attachment of the BCG to the urothelium and initiation of localized inflammation, which attracts innate immune cells that in turn draw cytotoxic T-lymphocytes (CTLs) and natural killer (NK) cells which attack the tumor cells [6, 18]. Bacteria can be engulfed by macrophages and dendritic cells (DCs), two types of antigen-presenting cells (APCs). Bacteria may also infect occasional residual cancer cells, which present bacterial protein fragments, attracting APCs that will ingest the infected host. Once a BCG-infected tumor cell has been ingested by an APC, its tumor antigens are now presented by the APCs, which further stimulate CTL proliferation, together with the bacterial infection itself. These effects upon CTL proliferation were not taken into account in the original BCG model [9].

Recent studies have described the BCG-immune system interactions: Biot et al. [18], Kikamura and Tsukamoto [19], and Bunimovich-Mendrazitsky et al. [12], which describe two distinct CTL populations capable of destroying tumor cells, either via tumor associated Ag mechanism or via bacterial associated Ag. Another important factor not accounted for in [9] is the rate of maturation and activation of CTL cells [20, 21]. A limiting factor of CTL development is the time required to identify bacterial and tumor antigens and to mobilize an effective immune response [22]. While the modeling of each relevant stage involved in this process is not possible within the current ODE system, a simplified model representing tumor-immune system interplay will provide insight to this fundamental yet complex interaction.

## 3. Description of the Model

Our model describes the parameters governing the efficacy of BCG treatment for bladder cancer. Based in part upon previous study [9], we have further optimized the model to account for the delay inherent to the immune response to BCG injection, taking into account the latest experimental findings regarding BCG immunotherapy of bladder cancer.

We model the stage in pathogenesis wherein no metastases have yet occurred, such that the entire dynamics of the system take place within the bladder lumen. Therefore, a one-site mathematical model is sufficient.

The BCG treatment model is composed of four nonlinear ODEs to characterize the interactions between the four different biological components, with the local quantity of each noted as follows:

- BCG bacteria within the bladder as *B*.
- Effector T-lymphocytes, principally CTLs that react to BCG and tumor antigens as *E*.
- Tumor cells infected with BCG as *T*_*i*_.
- Tumor cells not infected with BCG as *T*_*u*_.

To summarize briefly, we assume BCG to be introduced into the bladder at a constant rate *b*. The free BCG binds to tumor cells, infecting them at rate *p*_2_, with *μ*_1_ denoting the natural death rate of BCG. Tumor cells are tracked by a continuous variable, due to their large number, and the homogeneous nature of the tumor population. Effector cells (*E*) target and destroy infected and noninfected tumor cells (*T*_*i*_ and *T*_*u*_) at a rate *p*_3_ and take up BCG at a rate *p*_1_. Activation of the immune response is dependent upon:

1. the encounter between immune cells and BCG, controlled by parameter *p*_4_
2. the encounter between immune cells and tumor cells, controlled by parameter *α*.

The rate of inactivation of *E* cells via encounter with *T*_*i*_ is given by *p*_5_. Natural logistic tumor growth is characterized by a maximal growth rate coefficient *r* and is limited by the maximum number of tumor cells *K*. Finally, we denote by *μ*_2_ the natural death rate of effector cells. The equations describing the interactions between these four variables are given in the following system:

(1) B˙t=b−Btμ1+p1Et+p2Tut,E˙t=−μ2Et+p4Bt−p5Tit+αTut·Et−τt,T˙it=p2BtTut−p3TitEt−τt,T˙ut=Tut·−p2Bt+r1−TutK−p3Et−τt.

In system (1) in the second equation (effector cells $\dot{E}$ ) BCG induces the recruitment of the effectors with rate *p*_4_ *B*(*t*)*E*(*t* − *τ*(*t*)), while the tumor induces the recruitment of the effectors at a linear rate *αT*_*u*_(*t*)*E*(*t* − *τ*(*t*)), with delay function *τ*(*t*) = *τ*_0_ *e* ^−*λt*^, *λ* > 0. Correspondingly, eradication of infected *T*_*i*_ (in the third equation) and uninfected *T*_*u*_ (in the fourth equation) tumor cells by effector cells will produce with the same delay *τ*(*t*) function.

*τ*(*t*) is a time-varying function, representing the immune response delay in response to treatment, and expressing the number of effector cells in the cancer area. The delay is measured with reference to beginning of BCG treatment (*t* = 0), with a maximum delay of approximately 10 days. The influence of BCG tends towards zero over time, such that the delay function is similar to fading phenomenon: *τ*(*t*) = *τ*_0_ *e* ^−*λt*^.

## 4. Three Equilibria

The equilibria of system (1) are defined by the conditions $\dot{B}(t)=0$ , $\dot{E}(t)=0$ , ${\dot{T}}_{i}(t)=0$ , and ${\dot{T}}_{u}(t)=0$ that is equivalent to the system of four algebraic equations

(2) b=B∗μ1+p1E∗+p2Tu∗,p4B∗−p5Ti∗+αTu∗E∗=μ2E∗,p2B∗Tu∗=p3Ti∗E∗,−p2B∗+r1−Tu∗KTu∗=p3E∗Tu∗,

with three solutions:

(3) 1  B1∗=bμ1,E1∗=T1u∗=0,T1i∗=C≥0−arbitrary  nonnegative  constant.

(4) 2  B2∗=μ2p4,E2∗=p4b−μ1μ2p1μ2>0,T2i∗=T2u∗=0.E3∗=p2B3∗p3Ru,T3i∗=RuT3u∗,

(5) 3  B3∗=μ2+p5Ru−αT3u∗p4,E3∗=p2B3∗p3Ru,T3i∗=RuT3u∗,

where

(6) Ru=p1/p3p4FuGu+bFuHu−b,Fu=r1−T3u∗K,  Gu=αT3u∗−μ2,  Hu=μ1p2+1+p1p5p3p4T3u∗,

and *T*_3*u*_^*∗*^ is defined by the algebraic equation

(7) $$\begin{array}{c} p_{2}B_{3}^{*}\left(\;1+\frac{1}{R_{u}}\;\right)=F_{u}, \end{array}$$

with *B*_3_^*∗*^, *R*_*u*_, and *F*_*u*_ defined in (5) and (6) (see Appendix).

**Remark 1 .**

Putting *x* = *T*_3*u*_^*∗*^/*K*, *β* = *μ*_1_/*p*_2_, and *γ* = (1 + *p*_1_ *p*_5_/*p*_3_ *p*_4_)*K*, via (6), and a positivity of *R*_*u*_ for the third equilibrium one can obtain the estimation for *b*: *b* < *F*_*u*_ *H*_*u*_ = *r*(1 − *x*)(*β* + *γx*) ≤ *r*(*β* + *γ*)^2^/4*γ*.

## 5. Stochastic Perturbations, Centering, and Linearization

Let us assume that system (1) is exposed to stochastic perturbations that are of the white noise type and are directly proportional to the deviation of the system state (*B*(*t*), *E*(*t*), *T*_*i*_(*t*), *T*_*u*_(*t*)) from the point of equilibrium (*B* ^*∗*^, *E* ^*∗*^, *T*_*i*_^*∗*^, *T*_*u*_^*∗*^) and influence $\left(\dot{B}(t),\dot{E}(t),{\dot{T}}_{i}(t),{\dot{T}}_{u}(t)\right)$ immediately. In this way, system (1) takes the form

(8) B˙t=b−Btμ1+p1Et+p2Tut+σ1Bt−B∗w˙1t,E˙t=−μ2Et+p4Bt−p5Tit+αTuEt−τt+σ2Et−E∗w˙2t,T˙it=p2BtTut−p3TitEt−τt+σ3Tit−Ti∗w˙3t,T˙ut=Tut−p2Bt+r1−TutK−p3Et−τt+σ4Tut−Tu∗w˙4t,

where *σ*_*i*_ are constants and *w*_*i*_(*t*), *i* = 1,2, 3,4, are mutually independent standard Wiener processes. System (8) is a system of stochastic differential equations [17, 23]. Stochastic perturbations of a such type were first proposed in [24] and successfully used later in other researches (see [16, 17] and references therein). An important feature of this type of perturbations is that the equilibrium of the deterministic system is also a solution of a system with stochastic perturbations.

Let (*B* ^*∗*^, *E* ^*∗*^, *T*_*i*_^*∗*^, *T*_*u*_^*∗*^) be one from the equilibriums of system (8). Using (2) substitutes new variables *y*_1_(*t*) = *B*(*t*) − *B* ^*∗*^, *y*_2_(*t*) = *E*(*t*) − *E* ^*∗*^, *y*_3_(*t*) = *T*_*i*_(*t*) − *T*_*i*_^*∗*^, and *y*_4_(*t*) = *T*_*u*_(*t*) − *T*_*u*_^*∗*^, into system (8) for each equilibrium.

For the first equilibrium we obtain the nonlinear system

(9) y˙1t=−μ1y1t−B∗p1y2t+p2y4t−y1tp1y2t+p2y4t+σ1y1tw˙1t,y˙2t=−μ2y2t+p4B∗−p5Ti∗y2t−τt+p4y1t−p5y3t+αy4ty2t−τt+σ2y2tw˙2t,y˙3t=p2B∗y4t−p3Ti∗y2t−τt+p2y1ty4t−p3y2t−τty3t+σ3y3tw˙3t,y˙4t=r−p2B∗y4t+−p2y1t−ry4tK−p3y2t−τty4t+σ4y4tw˙4t.

Similarly for the second equilibrium

(10) y˙1t=−bB∗−1y1t−B∗p1y2t+p2y4t−y1tp1y2t+p2y4t+σ1y1tw˙1t,y˙2t=E∗p4y1t−p5y3t+αy4t−μ2y2t+μ2y2t−τt+p4y1t−p5y3t+αy4ty2t−τt+σ2y2tw˙2t,y˙3t=−p3E∗y3t+p2B∗y4t−p3y2t−τty3t+p2y1ty4t+σ3y3tw˙3t,y˙4t=r−p2B∗−p3E∗y4t+−p2y1t−ry4tK−p3y2t−τty4t+σ4y4tw˙4t,

and for the third one

(11) y˙1t=−bB∗−1y1t−B∗p1y2t+p2y4t−y1tp1y2t+p2y4t+σ1y1tw˙1t,y˙2t=E∗p4y1t−p5y3t+αy4t−μ2y2t+μ2y2t−τt+p4y1t−p5y3t+αy4ty2t−τt+σ2y2tw˙2t,y˙3t=p2Tu∗y1t−p3E∗y3t+p2B∗y4t−p3Ti∗y2t−τt+p2y1ty4t−p3y2t−τty3t+σ3y3tw˙3t,y˙4t=Tu∗−p2y1t−rK−1y4t−p3y2t−τt+−p2y1t−rK−1y4t−p3y2t−τty4t+σ4y4tw˙4t.

It is clear that stability of the zero solution of system (9) ((10), (11)) is equivalent to stability of the first (the second, the third) equilibrium of system (8).

**Remark 2 .**

To obtain sufficient conditions of stability in probability for nonlinear system with the order of nonlinearity higher than one it is enough [17] to get sufficient conditions of asymptotic mean square stability for the linear part of the considered nonlinear system.

Following Remark 2, represent the linear parts of systems (9) - (11) in the form

(12) $$\begin{array}{c} \dot{z}\left(\;t\;\right)=Az\left(\;t\;\right)+Dz\left(\;t-\tau \left(\;t\;\right)\;\right)+C\left(\;z\left(\;t\;\right)\;\right)\dot{w}\left(\;t\;\right), \end{array}$$

where *z* = (*z*_1_, *z*_2_, *z*_3_, *z*_4_)′, *w* = (*w*_1_, *w*_2_, *w*_3_, *w*_4_)′, *C*(*z*) = diag{*σ*_1_ *z*_1_, *σ*_2_ *z*_2_, *σ*_3_ *z*_3_, *σ*_4_ *z*_4_}, and the matrices *A*, *D* are, respectively, for the first equilibrium

(13) A=−μ1−p1B∗0−p2B∗0−μ200000p2B∗000r−p2B∗,D=00000p4B∗−p5Ti∗000−p3Ti∗000000,

for the second equilibrium

(14) A=−bB∗−1−p1B∗0−p2B∗p4E∗−μ2−p5E∗αE∗00−p3E∗p2B∗000r−p2B∗−p3E∗,D=00000μ20000000000,

and for the third equilibrium

(15) A=−bB∗−1−p1B∗0−p2B∗p4E∗−μ2−p5E∗αE∗p2Tu∗0−p3E∗p2B∗−p2Tu∗00−rTu∗K−1,D=00000μ2000−p3Ti∗000−p3Tu∗00.

## 6. Stability Investigation

For equations with delay there are two types of stability conditions: delay independent conditions and delay dependent conditions. Following the assumption that in the considered system (1) the delay is decreasing in infinity to zero, below we consider stability conditions that are delay independent. Nevertheless, the research method used here can be used also to obtain delay dependent stability conditions [17].

To investigate stability of the linear stochastic delay differential equations (12) consider the conditions for a matrix *A* = ‖*a*_*ij*_‖ to be the Hurwitz matrix.

**Definition 3 .**

The trace of the *k*−th order of a *n* × *n*-matrix *A* is defined as follows:

(16) $$\begin{array}{c} S_{k}=\underset{1\leq i_{1}<\cdots <i_{k}\leq n}{\sum }\left|\begin{array}{ccc} a_{i_{1}i_{1}} & \cdots & a_{i_{1}i_{k}}\\ \cdots & \cdots & \cdots \\ a_{i_{k}i_{1}} & \cdots & a_{i_{k}i_{k}} \end{array}\right|,\;k=1,\ldots ,n. \end{array}$$

Here, in particular, *S*_1_ = Tr(*A*), *S*_*n*_ = det(*A*), and *S*_*n*−1_ = ∑_*i*=1_^*n*^ *A*_*ii*_, where *A*_*ii*_ is the algebraic complement of the diagonal element *a*_*ii*_ of the matrix *A*.

**Lemma 4 (see [17]).**

A 3 × 3-matrix *A* is the Hurwitz matrix if and only if

(17) S1<0,S1S2<S3<0.

A 4 × 4-matrix *A* is the Hurwitz matrix if and only if

(18) S1<0,S1S2<S3<0,0<S12S4<S1S2−S3S3.

**Lemma 5 (see [17]).**

Put Φ_0_(*P*) = *A*′*P* + *PA* + diag{*p*_11_ *σ*_1_^2^, *p*_22_ *σ*_2_^2^, *p*_33_ *σ*_3_^2^, *p*_44_ *σ*_4_^2^}. If $\dot{\tau }(t)\leq 0$ and for some positive definite matrices *P* and *R* at least one of the inequalities,

(19) Φ0P+R+PDR−1D′P<0,Φ0P+R+D′PR−1PD<0,Φ0P+D′RD+PR−1P<0,

holds, then the zero solution of (12) is asymptotically mean square stable.

**Remark 6 .**

Via Schur complement (see Appendix) instead of the nonlinear Riccati type inequalities (19) one can use, respectively, the conditions with the following linear matrix inequalities (LMIs):

(20) Φ0P+RPDD′P−R<0,Φ0P+RD′PPD−R<0,Φ0P+D′RDPP−R<0.

**Remark 7 .**

For conditions (19) or (20) the matrix *A* has be the Hurwitz matrix; i.e., the conditions of Lemma 4 must be hold.

### 6.1. Stability of the First Equilibrium

Conditions (18) for the matrix *A* given in (13) do not hold since *S*_4_ = 0. But putting *T*_1*i*_ = 0 and rejecting the equation for ${\dot{T}}_{1i}$ we obtain system (12) for the equilibrium (*B*_1_^*∗*^, *E*_1_^*∗*^, *T*_1*u*_^*∗*^) = (*bμ*_1_^−1^, 0,0) with the 3 × 3 matrices

(21) A=−μ1−p1B1∗−p2B1∗0−μ2000r−p2B1∗,D=0000p4B1∗0000.

Here *S*_1_ = −(*μ*_1_ + *μ*_2_ + *p*_2_ *B*_1_^*∗*^ − *r*), *S*_2_ = *μ*_1_ *μ*_2_ + (*μ*_1_ + *μ*_2_)(*p*_2_ *B*_1_^*∗*^ − *r*), and *S*_3_ = −*μ*_1_ *μ*_2_(*p*_2_ *B*_1_^*∗*^ − *r*). It is easy to see that if *p*_2_ *B*_1_^*∗*^ > *r*, i.e., *bp*_2_ > *rμ*_1_, then conditions (17) hold; i.e., the matrix *A* given in (21) is the Hurwitz matrix.

Using [17] we obtain that the inequalities

(22) μ1>δ1,μ2>p4B1∗+δ2,p2B1∗>r+δ4,B1∗=bμ1,δi=12σi2,i=1,2,4

are sufficient conditions for asymptotic mean square stability of the zero solution of system (12), (21). From conditions (22) via Remark 2 the following statement follows.

**Lemma 8 .**

If

(23) μ1>δ1,μ1r+δ4p2<b<μ1μ2−δ2p4,

then the first equilibrium (*B*_1_^*∗*^, *E*_1_^*∗*^, *T*_1*u*_^*∗*^) = (*bμ*_1_^−1^, 0,0) of system (12) is stable in probability.

**Example 9 .**

Let *μ*_1_ = 1, *μ*_2_ = 0.41, *p*_2_ = 0.28, *p*_4_ = 0.12, *r* = 0.078. Then via (23) possible interval for *b* ∈ (0.279,3, 417). If *b* = 1 and *σ*_1_^2^ < 2, *σ*_2_^2^ < 0.58, *σ*_4_^2^ < 0.404, then the equilibrium (1,0, 0,0) is stable in probability.

### 6.2. Stability of the Second Equilibrium

For positivity of the second equilibrium it is necessary to suppose that *p*_4_ *b* > *μ*_1_ *μ*_2_. Calculating for system (12), (14) *S*_*i*_, *i* = 1,2, 3,4, via Definition 3, we obtain that conditions (18) hold:

(24) S1=−bB∗−1+μ2+p3E∗+p2B∗+p3E∗−r=−2.2889<0,S2=bB∗−1+p3E∗μ2+p1p4B∗E∗+bB∗−1·p3E∗+bB∗−1+μ2+p3E∗p2B∗+p3E∗−r=1.6494>0,S3=−μ2p3E∗+bB∗−1p3E∗+μ2bB∗−1+p1p4B∗E∗p2B∗+p3E∗−r+μ2p3bB∗−1+p1p3p4B∗E∗2=−0.8115<0,S4=p2B∗+p3E∗−rμ2bB∗−1+p1p4B∗E∗·p3E∗=3.0938∗10−5>0,S1S2−S3=−2.9639<0,S12S4=16.2086∗10−5<2.4052=S1S2−S3S3.

Note however that two last equations of this system can be considered separately. So, if

(25) p3E∗>δ3,p2B∗+p3E∗>r+δ4,B∗=μ2p4,E∗=p4b−μ1μ2p1μ2>0,

or

(26) b>μ2p4μ1+p1p3δ,where  δ=max⁡δ3,δ4+r−μ2p4p2,

then the zero solution of two last equations of system (12), (14) is asymptotically mean square stable [17].

From this it follows that for stability investigation of the second equilibrium it is enough to investigate asymptotic mean square stability of system (12) with

(27) A=−bB∗−1−p1B∗p4E∗−μ2,D=000μ2,Cz=σ1z1t00σ2z2t.

For this aim one can solve the nonlinear inequalities (19) or to check feasibility of the LMIs (20) via MATLAB.

**Example 10 .**

Let *μ*_1_ = 1, *μ*_2_ = 0.41, *p*_1_ = 1.25, *p*_2_ = 0.28, *p*_3_ = 1.1, *p*_4_ = 0.12, *p*_5_ = 0.003, *r* = 0.078, and *α* = 3.7. The second equilibrium exists if *b* > *μ*_1_ *μ*_2_ *p*_4_^−1^ = 3.4167. Checking the LMIs (20) for system (12), (27) we obtain that in deterministic case (*σ*_1_ = *σ*_2_ = 0) the zero solution of the system is asymptotically stable for *b* ≥ 3.417. In the stochastic case for *σ*_1_ = 0.45, *σ*_2_ = 0.31 the zero solution of system (12), (27) is asymptotically mean square stable for *b* ≥ 3.896. It means, for example, that for *b* = 3.896 the equilibrium (*B*_2_^*∗*^, *E*_2_^*∗*^, *T*_2*i*_^*∗*^, *T*_2*u*_^*∗*^) = (3.4167,0.1122,0, 0) is stable in probability.

Checking the LMIs (20) for the initial system (12), (14) we obtain the close result: in the deterministic case the zero solution of system (12), (14) is asymptotically stable for *b* ≥ 3.417. In the stochastic case for *σ*_1_ = 0.45, *σ*_2_ = 0.31, *σ*_3_ = 0.5, and *σ*_4_ = 1.4 the zero solution of system (12), (14) is asymptotically mean square stable for *b* ≥ 3.902. Conditions (18) and (26) in this case naturally hold. For example, for *b* = 3.902 the equilibrium (*B*_2_^*∗*^, *E*_2_^*∗*^, *T*_2*i*_^*∗*^, *T*_2*u*_^*∗*^) = (3.4167,0.1136,0, 0) is stable in probability.

**Remark 11 .**

Note that via (23), (26), for stability of the first equilibrium must be *rμ*_1_/*p*_2_ < *b* < *μ*_1_ *μ*_2_/*p*_4_ and for stability of the second equilibrium must be *b* > *μ*_1_ *μ*_2_/*p*_4_.

### 6.3. Stability of the Third Equilibrium

**Example 12 .**

Put *μ*_1_ = 1, *μ*_2_ = 0.41, *p*_1_ = 1.25, *p*_2_ = 0.28, *p*_3_ = 1.1, *p*_4_ = 0.12, *p*_5_ = 0.003, *α* = 3.7, *K* = 80, *r* = 0.078, and *b* = 0.99. By given values of the parameters via (6), (7) we obtain *T*_3*u*_^*∗*^ = 11.19, *F*_*u*_ = 0.0671, *G*_*u*_ = 40.993, *H*_*u*_ = 15.0793, and *R*_*u*_ = 1247.6 and via (5) the equilibrium is (*B* ^*∗*^, *E* ^*∗*^, *T*_3*i*_^*∗*^, *T*_3*u*_^*∗*^) = (7.4126,0.0015,13961,11.19). Checking LMIs (20) we can conclude instability of this equilibrium even in the deterministic case. Similar calculations for different *b* < 1.7466 (via Remark 1) show that the third equilibrium is unstable.

## 7. Conclusion

In this work we present the improved model of BCG immunotherapy in superficial bladder cancer. This study investigates stability of new treatment model with constant instillations of BCG under stochastic perturbations. The model demonstrates several stable states which depend on biologically related parameters and initial conditions.

The innovation of our study is the involvement of CTL cells, specific for the tumor antigen due to BCG infection. Adding BCG to the tumor-immune interaction may increase the immune response, which most benefit from the addition of the antigen. These effector cells capture tumor cells after time delay which has been used for maturation of these effector cells. The entire reaction can take place only with the presence of BCG. We added new terms in two equations (the second and the forth) in system (1). The new terms stand for the killing of uninfected tumor cells by effector cells.

It is shown that the considered system has three equilibria describing the different states of the patient. Stability of these states under stochastic perturbations which are directly proportional to the deviation of the patient's current state from the equilibrium state is investigated. New sufficient conditions for stability in probability of two equilibria and instability of the third equilibrium were obtained using the theoretical method of Lyapunov functionals and the numerical method of linear matrix inequalities (LMIs).

In the first equilibrium we obtain weak immune response because *E*_1_^*∗*^ = 0. In addition BCG-infected tumor cells were remained (*T*_1*i*_^*∗*^ > 0); that indicates cancer will not obligatorily be eradicated, although this equilibrium is stable. In the second equilibrium we receive *T*_2*i*_^*∗*^ = *T*_2*u*_^*∗*^ = 0; that means the cancer could be eradicated after BCG immunotherapy with dose *b* > (*μ*_2_/*p*_4_)(*μ*_1_ + (*p*_1_/*p*_3_)*δ*). This equilibrium has very strong immune response *E*_2_^*∗*^ > 0 and it is stable.

The third equilibrium is unstable.

The dynamic behavior of the system investigated from the point of view of local stability and a detailed analysis on stability of equilibrium was examined. As proposed in the paper research methods allow continuing and specifying investigation of the considered model and getting its new useful properties.

However, we have to note that this study has following limitations:

1. This model takes into account 3-biological processes (tumor, immune system, and BCG interactions) only, which are captured by 4 differential equations.
2. We examine continued BCG treatment with a constant rate *b*, although in a real life pulsed therapy and variable rate can be used.
3. The duration of BCG treatment is not time limited in our model.

By capturing the main parameters of the BCG, maximum tumor size, tumor growth rate, and immune response parameters, we create a silicone model and develop an algorithm for eliminating cancer. We would like to raise awareness in the community of urological-oncological doctors about the possibilities of mathematical modeling and receive quantitative data to improve this model. The ability to plan and predict by calculating a modulated dose of treatment can benefit patients who are unable to take routine treatment because of its serious side effects, as well as to patients who were previously considered not to respond.

## Data Availability

The data used to support the findings of this study are available from the corresponding author upon request.

## Appendix

*The Third Equilibrium.* Using *T*_3*i*_^*∗*^ = *R*_*u*_ *T*_3*u*_^*∗*^, from the second and the third equations (2), we obtain (5). Substituting (5) into the last equation (2), we have

(A.1)
$$\begin{array}{c} r\left(\;1-\frac{T_{3u}^{*}}{K}\;\right)=p_{2}B^{*}\left(\;1+\frac{1}{R_{u}}\;\right). \end{array}$$

Using (5), (6), and the representation *p*_4_ *B* ^*∗*^ = *p*_5_ *R*_*u*_ *T*_3*u*_^*∗*^ − *G*_*u*_, from the first equation (2), we obtain

(A.2)
b=B∗μ1+p2T3u∗+p1p2B∗p3Ru=B∗μ1+p2T3u∗+p1p2p3p4Rup5RuT3u∗−Gu=p2B∗Hu−p1Gup3p4Ru.

From (A.1), (A.2), the representation (6) for *R*_*u*_ and (7) for *T*_3*u*_^*∗*^ follow.

*Schur Complement.* The symmetric matrix $\left[\begin{array}{cc} A & B\\ B^{\prime } & C \end{array}\right]$ is negatively definite if and only if *C* and *A* − *BC* ^−1^ *B*′ are both negatively definite.
